# Polymyositis as a manifestation of chronic graft‐versus‐host disease after allo‐HSCT

**DOI:** 10.1002/ccr3.1709

**Published:** 2018-07-12

**Authors:** Lijuan Meng, Suqiong Ji, Qiong Wang, Bitao Bu

**Affiliations:** ^1^ Department of Neurology Tongji Hospital Tongji Medical College Huazhong University of Science and Technology Wuhan China

**Keywords:** allogeneic haematopoietic stem cell transplantation, graft‐versus‐host disease, immunotherapy, pathology, polymyositis

## Abstract

Patients who received allogeneic haematopoietic stem cell transplantation (allo‐HSCT) may develop T cell‐mediated immunologic injury to muscles, recapitulating the characteristics of polymyositis clinically and pahtologically. Polymyositis is a rarely reported complication of graft‐versus‐host disease (GVHD), which often responds well to corticosteroids and immunosuppressive treatment.

## 1 INTRODUCTION

Polymyositis (PM) is a rare neuromuscular presentation of chronic graft‐versus‐host disease (cGVHD) in patients who underwent allogeneic hematopoietic stem cell transplantation (allo‐HSCT).^1^, ^2^ Here, we reported two cases of cGVHD associated‐polymyositis after allo‐HSCT, successfully treated with immunotherapy.

## 2 CASE REPORTS

### 2.1 Patient 1

A 48‐year‐old woman was diagnosed with acute myeloid leukemia (AML) in 2013,who fortunately achieved a complete remission of bone marrow after two courses of chemotherapy (IA regimen: idarubicin hydrochloride + cytarabine). Allogeneic HSCT deriving from her HLA‐identical brother was performed in February 2014 after a reduced intensity conditioning regimen (Ara‐c/Bu/Cy/Me‐CCNU). Graft‐versus‐host disease prophylaxis included cyclosporine(50 mg BID, and then gradually reduced the dose to 25 mg BID)and dexamethasone (5 mg QD). Ten months later, she began to complain limb weakness and mild dyspnea after walking. No myalgia, skin rash, or obvious dysphagia was documented. She stopped taking cyclosporine in May 2015. The muscle weakness and dyspnea were significantly aggravated after a fever in July 2015. The proximal muscles [Medical Research Council (MRC)graded 3] were more severely affected than the distal ones(MRC graded 4). Obvious muscle atrophy was evident on the proximal muscles. Deep tendon reflexes were slightly decreased. The somatic sensations were normal.

**Figure 1:**
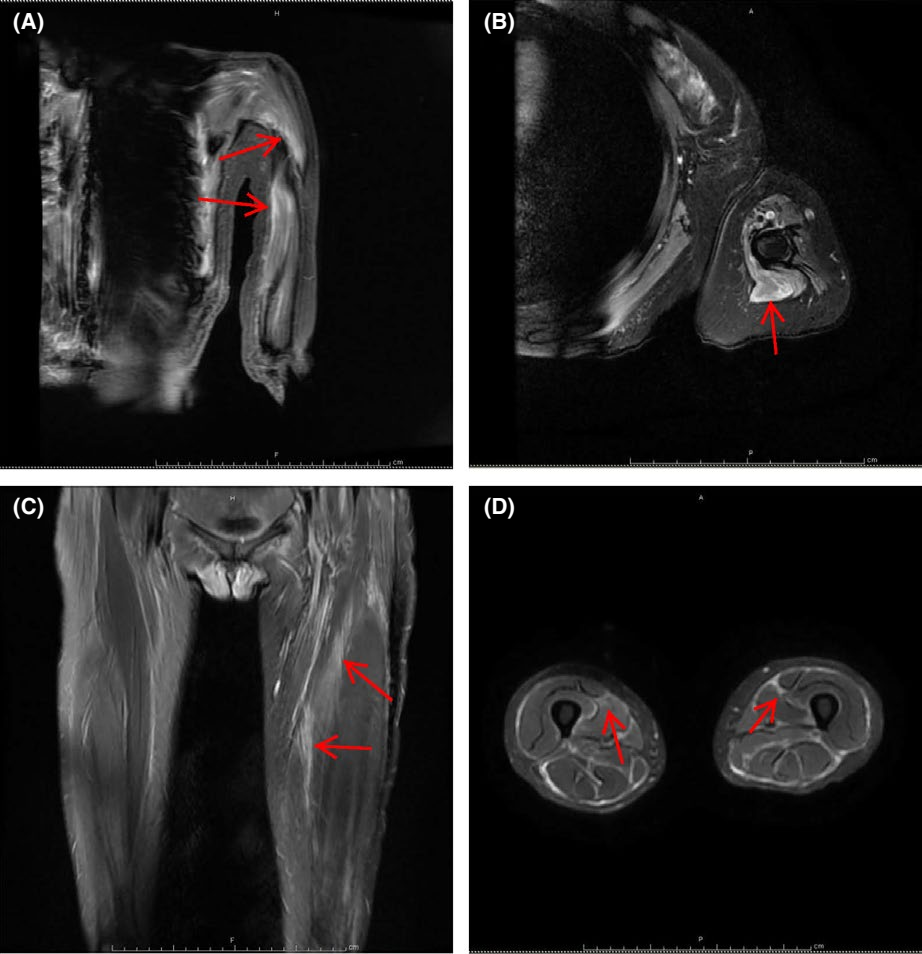
MRI Imaging. T2‐weighed Imaging Showed High Intensity in the Biceps, Deltoid, and Triceps Muscles (A & B from the CASE 1), and in Muscles and Fascia of the Thigh (C & D from the Case 2)

Blood analyses disclosed that the levels of creatine kinase (CK), lactate dehydrogenase (LDH),aspartate aminotransferase (AST), alanine aminotransferase (ALT), and myoglobin were elevated. C‐reactive protein (CRP), blood routines, and thyroid glands were in normal ranges and the antinuclear antibodies, and myositis‐specific autoantibodies were not detected (Table 1). Electromyography (EMG) displayed the myopathic changes. Magnetic resonance imaging (MRI) showed areas of abnormally high signal intensity in the muscles of the left arm on fat‐suppressed T2‐weighed image, especially the biceps, deltoids, triceps, and subscapular muscles (Figure 1A,B), suggesting muscle inflammation and edema.

**Table 1:** Selected hematologic and immunological test results for Patients 1 and 2

	Ref range	Patient 1	Patient 2
Creatine kinase (U/L)	3‐170	2118	18
Lactate dehydrogenase (U/L)	135‐214	528	241
Aspartate aminotransferase (U/L)	4‐32	89	192
Alanine aminotransferase (U/L)	4‐33	85	137
Myoglobin (ng/mL)	1.0‐140.1	1057.90	11.3
C‐reactive protein (mg/L)	0.3‐3.0	0.2	1.6
Antinuclear antibodies	Negative	1:100	1:320
Myositis autoantibody	Negative	Negative	Negative
Thyroid glands	Negative	Negative	Negative

**Figure 2:**
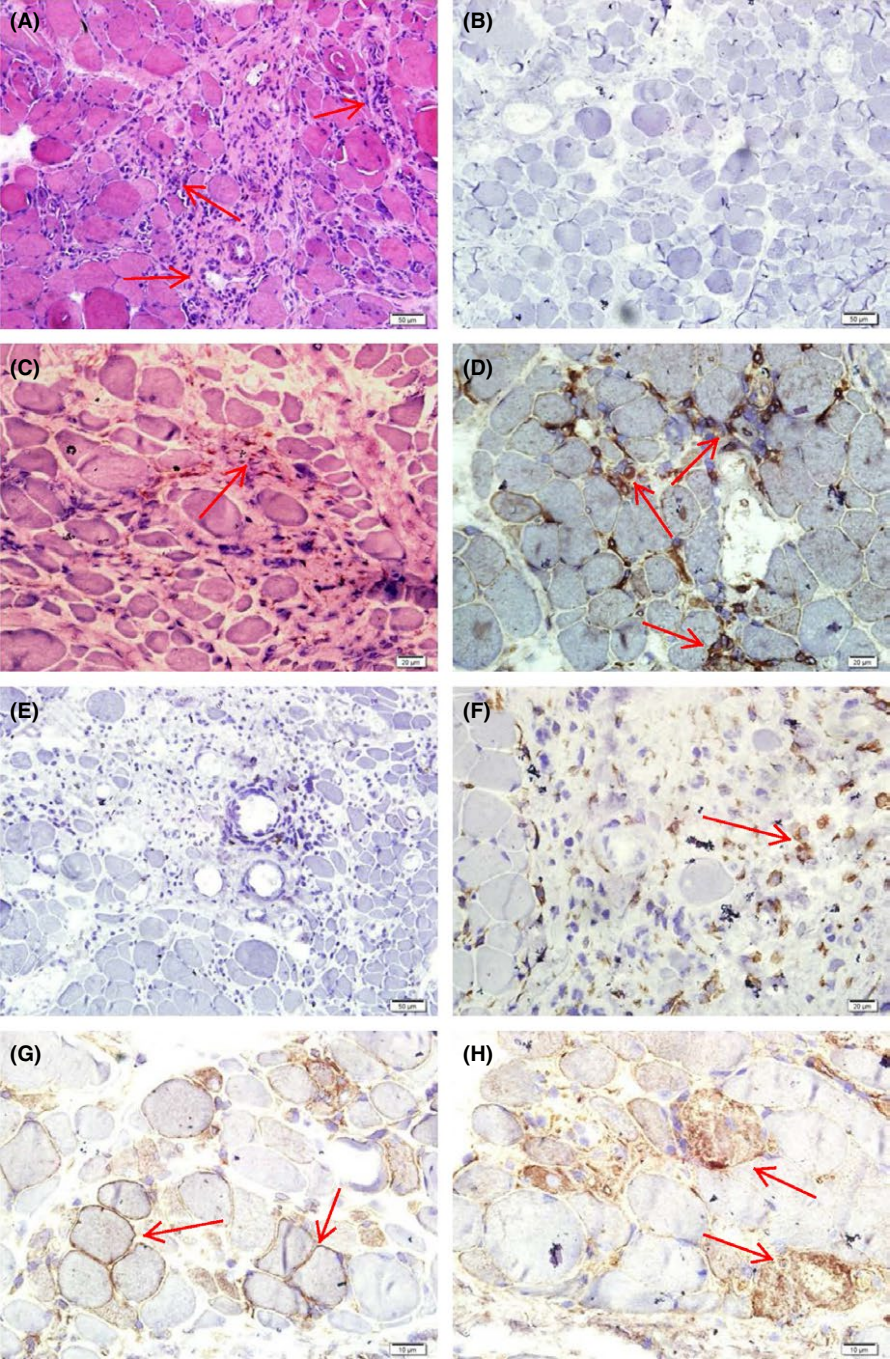
Pathological Findings. A, H&E: Prominent inflammatory cell infiltration with degenerated and necrotic myofibers; scale bar = 50 μm. B‐H, Immunohistochemical staining revealed that the infiltrated lymphocytes were predominantly T cells while B lymphocytes were negative. B, Control; scale bar = 50 μm. Immunostaining of CD4 (C, scale bar = 20 μm), CD8 (D, scale bar = 20 μm), CD20 (E, scale bar = 50 μm). F, CD68+ macrophages accumulated in the necrotic myofibers; scale bar = 20 μm. G, Major histocompatibility complex class I (MHC‐1) was positive in myofiber membranes; scale bar = 10 μm. H, The membrane attack complex (MAC) deposited in the necrotic myofibers; scale bar = 10 μm

Pathological study of the biopsy specimens from the left biceps demonstrated prominent inflammatory cell infiltration with degenerated and necrotic myofibers. Immunohistochemical staining of the sections revealed that the infiltrating lymphocytes were predominantly T cells (mainly CD8+ T cells with a few CD4+ cells), while B lymphocytes were scarce (no CD20+ cells). Major histocompatibility complex class I (MHC‐1) was upregulated in some myofibers. In addition, the membrane attack complex (MAC) was deposited in the necrotic myofibers (Figure 2).

Based on the clinical presentation and the pathologic findings on the biopsied muscle, the polymyositis after allo‐HSCT was considered. The patient was initially treated with an increased dosages of prednisone and cyclosporin A (CsA), but no significant improvement was achieved. Thus, rituximab (100 mg) plus a low dose of intravenous immunoglobulin (IVIG, 5‐10 g) weekly was applied for consecutive 6 weeks, the patient finally achieved a remarkable improvement of her symptoms with a normal CK level. Until now, the patient has been followed up for one and a half years, and she remained symptom‐free on oral prednisone and CsA.

### 2.2 Patient 2

A 31‐year‐old woman was diagnosed with AML 20 months ago. A first complete remission of AML was achieved with two courses of chemotherapy (DA regimen: pirarubicin + cytarabine; cytarabine + methylprednisolone). Fifteen months ago, she received allo‐HSCT after a reduced intensity conditioning regimen with IDA/Bu/Fu/Me‐CCNU. The postoperative regimen was consisted of cyclosporine (25 mg BID) and methylprednisolone (40 mg QD and then gradually reduced the dose to 20 mg QD) for GVHD prophylaxis. She was hospitalized because she had limb weakness and myalgia for 5 months. Five months ago, she felt myalgia in both the lower limbs (MRC graded 4) and later the upper limbs (MRC graded 4‐). The muscles on the four extremities were generally tender and slight atrophic. Deep tendon reflexes were slightly decreased. The sensation was normal.

Laboratory tests showed the normal ranges of CK and myoglobin levels. The myositis‐specific autoantibodies were not positively detected. The antinuclear antibody (ANA) titer was weakly positive at 1:320 (Table 1). MRI showed high intensities in fat‐suppressed T2‐weighed image in the supraspinatus, scapular muscles, arm muscle group, as well as the leg muscle group and fascia (Figure 1,C,D). EMG detected myopathic changes. Muscle biopsy of the quadriceps femoris revealed that the degeneration and necrosis of myofibers were evident, with a large number of inflammatory cell infiltrates in the endomysium, similar to those described in the biopsied muscle from the case one.

The therapy was shifted from the previous regimen consisting of oral prednisone and CsA to tacrolimus (3 mg QD) combined with oral methylprednisolone (40 mg QD). The symptoms began to relieve in 2 weeks and had disappeared at about 3 months after the new regimen applied. She had been followed up for over 1 year and was in a good condition on the daily dosage of tacrolimus at 3 mg with methylprednisolone at 12 mg.

## 3 DISCUSSION

Polymyositis (PM) is a rare neuromuscular manifestation of chronic GVHD in patients who received allo‐HSCT.^1^, ^2^ The two cases in our study presented an insidious onset and a slowly progressive mode. The clinical presentation, elevation of CK and/or LDH, myopathic changes in EMG, the abnormal signals on muscular MRI, and the pathological findings in the affected muscles confirmed the diagnosis of PM.^3^ That the autoantibodies implying idiopathic inflammatory myopathies, connective tissue diseases or para‐neoplastic syndromes were not detected ruled out the possibilities of other diseases.

In order to reduce the occurrence of GVHD, a reduced intensity conditioning regimen before allo‐HSCT is often necessary.^4^ Although the two cases had received both the standardized pretreatment and sustained immunosuppressants after allo‐HSCT, the infused donor's lymphocytes still triggered immune attacks to the host via T‐cell pathway.^2^, ^5^ Theoretically, the clonally expanded CD8 +  cytotoxic T cells invaded the muscle fibers which overexpress MHC class I antigens, in turn led to myofiber necrosis via the perforin pathway^6^ and activated complement system.^5^ The immunohistochemical staining suggested that the cellular dysimmunity played a key role in the development of GVHD‐associated myositis in the two cases. Activation of complement system might have contributed to the pathogenesis since the necrotic myofibers were positive for MAC, which is a marker for complement system activation.^7^, ^8^, ^9^

The fact that the case responded well to Rituximab plus small dosage IVIG, signifies that the humoral dysimmunity plays an important role in the development of myositis in addition to cellular autoimmunity, because Rituximab blocks the generation of autoantibodies from B cells.^10^ Although no B lymphocytes were infiltrated in the necrotic myofibers in the two cases, the possibility that the activated circulating B cells may act in harmony with T cells and activate complement system to destroy the myofibers is substantiated by the satisfactory responsiveness to Rituximab.

In conclusion, we emphasize that the patients who received allo‐HSCT may develop T cell‐mediated immunologic injury to muscles which is similar to polymyositis in terms of clinical features and myopathologic changes. Polymyositis as a presentation of chronic GVHD often responds well to corticosteroids and immunosuppressive treatment. Targeting both the T and B cells is effective.

## CONFLICT OF INTEREST

No conflict of interests were declared.

## AUTHORSHIP

LM: Collected data, wrote the original draft. BB: performed supervision and correction of the text. SJ: contributed to collecting data. QW: carried out preparation of pathology slides.
